# Inhibitory Effect of the Melanocortin Receptor Agonist Melanotan-II (MTII) on Feeding Depends on Dietary Fat Content and not Obesity in Rats on Free-Choice Diets

**DOI:** 10.3389/fnbeh.2015.00358

**Published:** 2015-12-24

**Authors:** José K. van den Heuvel, Leslie Eggels, Andrea J. van Rozen, Eric Fliers, Andries Kalsbeek, Roger A. H. Adan, Susanne E. la Fleur

**Affiliations:** ^1^Department of Endocrinology and Metabolism, Academic Medical Center, University of AmsterdamAmsterdam, Netherlands; ^2^Department of Neuroscience and Pharmacology, Rudolf Magnus Institute of Neuroscience, Utrecht University Medical CentreUtrecht, Netherlands; ^3^Hypothalamic Integration Mechanisms, Netherlands Institute for NeuroscienceAmsterdam, Netherlands

**Keywords:** melanocortin, obesity, diets, food intake, saturated fat, liquid sugar

## Abstract

**Introduction:** Conflicting data exist on sensitivity changes of the melanocortin system during diet-induced obesity. We hypothesized that melanocortin sensitivity depends on diet composition, in particular on the fat content rather than the level of obesity. The aim of this study was to determine the influence of diet composition on feeding responses to a melanocortin receptor agonist, using free-choice diets that differ in food components.

**Methods:** Male Wistar rats were subjected to a chow (CHOW) diet or a free-choice (fc) diet of either chow, saturated fat and liquid sugar (fcHFHS), chow and saturated fat (fcHF), or chow and liquid sugar (fcHS) for 4 weeks. Melanocortin sensitivity was tested by measuring food intake following administration of the melanocortin 3/4 receptor agonist melanotan II (MTII) or vehicle in the lateral ventricle. In a separate experiment, proopiomelanocortin (POMC) and agouti-related protein (AgRP) mRNA levels were determined in the arcuate nucleus with *in situ* hybridization in rats subjected to the free-choice diets for 4 weeks.

**Results:** Rats on the fcHFHS diet for 4 weeks show increased caloric intake and body weight gain compared to rats on the CHOW, fcHS and fcHF diet. Caloric intake and body weight gain was comparable between rats on the fcHF, fcHS, and CHOW diet. After 4 weeks diet, POMC and AgRP mRNA levels were not different between diet groups. MTII inhibited caloric intake to a larger extent in rats on the fcHF diet compared to rats on the CHOW, fcHFHS or fcHS diet. Moreover, the fat component was the most inhibited by MTII, and the sugar component the least.

**Conclusion:** Rats on the fcHF diet show stronger food intake inhibition to the melanocortin receptor agonist MTII than rats on the CHOW, fcHS, and fcHFHS diet, which is independent of caloric intake and body weight gain. Our data point toward an important role for diet composition, particularly the dietary fat content, and not obesity in the sensitivity of the melanocortin system.

## Introduction

Energy homeostasis is regulated by the brain through a complex neural network with an important role for the arcuate nucleus of the hypothalamus. Within this neural network the melanocortin system plays a critical role in maintaining stable energy balance. Separate neuron populations within the arcuate nucleus express the precursor of the melanocortin receptor agonist proopiomelanocortin (POMC) and the melanocortin receptor inverse agonist agouti-related protein (AgRP). Two melanocortin receptors (MC3R and MC4R) are expressed in the brain, although both have been implicated in the regulation of energy balance, MC4R has a predominant role in the regulation of food intake (Adan et al., 2006).

A large number of studies has shown that central administration of alpha-MSH and Melanotan II (MTII), a non-selective melanocortin 3/4 receptor agonist, strongly reduces food intake and increases energy expenditure. Additionally, a large number of studies have investigated the effects of MC ligands on macronutrient intake and most studies [but not all (Bauer et al., 2009; Panaro and Cone, 2013)] link melanocortin signaling with preference for fat intake in mice (Koegler et al., 1999; Butler et al., 2001; Samama et al., 2003), rats (Hagan et al., 2001; Tracy et al., 2008; Mul et al., 2012), and humans (van der Klaauw et al., 2015).

Additionally, in studies investigating the sensitivity of the melanocortin system during diet-induced obesity conflicting data have been described, as obese animals show both decreased (Clegg et al., 2003) and increased (Hansen et al., 2001) food intake responses to melanocortin receptor ligands, compared to lean animals. The reason for this discrepancy remains unknown, but may be due to the diet composition (Kinzig et al., 2005; Morens et al., 2005), and particularly on the consumption of fat (Clegg et al., 2003). Moreover, the combination of studying melanocortin system sensitivity (using melanocortin receptor ligands) during obesity and macronutrient intake has not been performed before.

To distinguish between the effect of obesity and diet composition, we use a diet with separate food components, rather than pellet diets that differ in fat and sugar content. In this free-choice diet model rats receive different free-choice diets that consist of separate food components, i.e., saturated fat, liquid sugar or both, in addition to the standard diet with chow and tap water (CHOW). Using this unique free-choice diet model, we aimed to investigate whether the reduction of food intake by melanocortins depends on diet composition, obesity or both. To determine the influence of diet composition on the melanocortin system in rats on the free-choice diets, we measured POMC and AgRP mRNA levels in the arcuate nucleus and determined feeding responses to MTII on total caloric intake and separate diet components after 4 weeks diet.

## Materials and Methods

### Animals and Dietary Intervention

Male Wistar rats (250–280 g) were individually housed in a temperature (21–23°C) and light-controlled room (lights on 0700–1900). The diets used in this study comprised three free-choice diets in which rats could unrestrictedly eat any of the diet components: (1) Free-choice high-fat high-sugar diet (fcHFHS): a dish of saturated fat [Beef tallow (Ossewit/Blanc de Boeuf), Vandemoortele, Belgium], a bottle of 30% sugar water (1.0 M sugar mixed from commercial grade sugar and tap water), normal standard chow [special diet service (SDS), England] and normal tap water. (2) Free-choice high-fat diet (fcHF): a dish of saturated fat in addition to normal standard CHOW. (3) Free-choice high-sugar diet (fcHS): a bottle of 30% sugar water in addition to normal standard CHOW. The rats on the control diet remained on normal standard CHOW. Body weight and 24 h caloric intake (kcal) were monitored at least once a week. Total caloric intake was the sum of each individual food component of which the caloric intake was determined as follows: chow: 3.31 kcal/g; fat: 9 kcal/g and sugar solution: 1.2 kcal/g. Animal experiments were approved by the Committee for Animal Experimentation of the Academic Medical Center of the University of Amsterdam.

### Surgery and Procedure for Intracerebroventricular (ICV) Injections

Rats were implanted with a permanent 22-gage guide cannula (Plastics One, Bilaney, Germany) placed into the lateral ventricle (coordinates 0.8 mm posterior from bregma, 1.4 mm lateral from midline, 5.0 mm below the surface of the brain). Guide cannulas were secured to the skull using three anchor screws and dental cement and occluded by a 28-gage stainless steel dummy cannula (Plastics One, Bilaney Consultants GmbH, Düsseldorf, Germany) extending 0.5 mm beyond the guide. Immediately after surgery, rats received an analgesic subcutaneously (Carprofen, 0.5 mg/100 g BW) and were housed individually. After recovery from surgery, rats received a vehicle injection and 2 days later were randomly assigned to either of the diet groups (CHOW, fcHS, fcHF, or fcHFHS) and were maintained on their respective diets throughout the remainder of the experiment. All ICV injections were delivered in a volume of 3 μl manually with a Hamilton syringe over 45 s. Cannula placement was checked by inspecting post-mortem thionin-stained brain sections under a low-power microscope.

### Feeding Responses to MTII

After 4 weeks on either diet [CHOW (*n* = 5), fcHS (*n* = 5), fcHF (*n* = 5), or fcHFHS (*n* = 6)] rats received an ICV injection of 0.3 nmol Melanotan II acetate salt (MTII; Sigma–Aldrich, Netherlands, M8693) or vehicle (PBS). On the experimental day, all food components except water were removed and weighed at the beginning of the light phase (10AM). Between 2 and 1 h before the dark phase, rats were injected with either MTII or vehicle in randomized order using the same procedure as described previously (van den Heuvel et al., 2014b). MTII was administered prior to the dark phase in order to measure an inhibition of physiological food intake. Food was returned at lights off and food intake was measured 15 h later, in order to determine the food consumption during their natural eating period. Each rat received MTII in counterbalanced order, separated by a week, due to the long-lasting anorectic effects of MTII.

### POMC and AgRP *in Situ* Hybridization

In a separate study, using the same dietary protocol, rats were subjected again to CHOW (*n* = 7), fcHS (*n* = 7), fcHF (*n* = 7), or fcHFHS (*n* = 7) for 4 weeks. Food intake and body weight were monitored at least once a week. After 4 weeks rats were decapitated between 0900 and 1000 and brains were quickly frozen on dry ice and used for *in situ* hybridization. Coronal sections of 20 μm were labeled with 33P antisense RNA probes for POMC and AgRP mRNA according to the protocol previously described (van den Heuvel et al., 2014a). The films were developed and POMC and AgRP expression levels in arcuate nucleus were quantitatively analyzed using an Epson-Perfection 4990 Photo-flatbed-scanner. All images (800 dpi) were analyzed using ImageJ (Rasband, WS, NIH, Bethesda, MD, USA, http://rsbweb.nih.gov/ij/ 1997–2005). Gray values were determined in regions of interest and measured bilaterally. Specific signal was calculated by the subtraction of the background value.

### Statistical Analysis

All results are presented as means ± SEM. One-way analysis of variance (ANOVA) was performed to determine the difference in body weight, adiposity, caloric intake and gene expression levels. If the ANOVA was significant, *post hoc* analysis was performed to detect individual group differences (Tukey). Feeding responses to MTII were first analyzed using two-way ANOVA to determine effects of drug and diet. When significant, percentage suppression from baseline was calculated and data were analyzed by one-way ANOVA when groups were compared. If the AVOVA was significant, *post hoc* analysis was performed to detect individual group differences (Tukey). Significance was set at *P* < 0.05.

## Results

### Energy Balance

After 4 weeks diet caloric intake in fcHFHS rats was significantly higher compared to rats on CHOW, fcHS, and fcHF diet [*F*(3,20) = 21,17; *P* < 0.01] (**Table 1**). Four weeks body weight gain was increased in rats on fcHFHS diet compared with rats on CHOW, fcHS, and fcHF diet [*F*(3,20) = 5.08; *P* < 0.05] (**Table 1**). Epididymal fat mass accumulation was significantly increased in rats on the fcHS, fcHF, and fcHFHS diets compared to rats on the CHOW diet. Moreover, fat mass in rats on the fcHFHS diet was significantly higher compared to rats on the fcHS and fcHF diets [*F*(3,20) = 13,65; *P* < 0.001] (**Table 1**).

**Table 1:** Characteristics of rats on different free-choice diets.

	CHOW	fcHS	fcHF	fcHFHS
*n*	5	5	5	6
Body weight (g)	387 ± 14^a^	395 ± 8^a^	401 ± 13^a^	423 ± 11^b^
Body weight gain (g)	54.0 ± 5.2^a^	61.6 ± 5.4^a^	59.0 ± 3.8^a^	86.2 ± 5.7^b^
Epididymal white adipose tissue (g)	2.03 ± 0.1^a^	3.17 ± 0.2^b^	3.29 ± 0.3^b^	4.40 ± 0.3^c^
Caloric intake, kcal/day	80.4 ± 3.7^a^	83.2 ± 5.5^a^	89.8 ± 3.7^a^	111.1 ± 3.6^b^
Chow (kcal/day)	80.4 ± 3.7	36.6 ± 2.9	40.7 ± 4.6	44.6 ± 4.8
Sugar (kcal/day)	-	46.6 ± 3.2	-	36.8 ± 3.3
Fat (kcal/day)	-	-	49.1 ± 5.5	29.7 ± 3.4
% chow	100	43.9 ± 3.6	50.4 ± 7.7	40.2 ± 3.6
% sugar	-	56.1 ± 2.2	-	33.1 ± 2.3
% fat	-	-	49.6 ± 3.3	26.7 ± 1.9
Caloric content (kcal/g)	3.1	2.1	6.1	4.9
% fat in diet	4	1.8	56.5	28.3
% carbohydrate in diet	75	89	34	63.2
% protein in diet	21	9.3	9.5	8.4


Values are mean ± SEM. Different letters represent significant differences between groups (*P* < 0.05).

### Arcuate Nucleus POMC and AgRP Expression Levels

After 4 weeks diet, POMC and AgRP mRNA levels in the arcuate nucleus of the hypothalamus were determined in rats on CHOW, fcHS, fcHF, and fcHFHS diet. No significant differences were found between the diet groups for POMC mRNA levels ANOVA [*F*(3,27) = 0.27; *P* = 0.85]; CHOW 100 ± 8, fcHS 107 ± 10, fcHF 98 ± 4, fcHFHS 102 ± 8 in arbitrary units and as percentage of CHOW. No significant differences were found between the diet groups for AgRP mRNA levels ANOVA [*F*(3,27) = 0.57; *P* = 0.64]; CHOW 100 ± 11, fcHS 101 ± 12, fcHF 102 ± 13, fcHFHS 84 ± 7 in arbitrary units and as percentage of CHOW.

### Feeding Response to Central MTII

**FIGURE 1:**
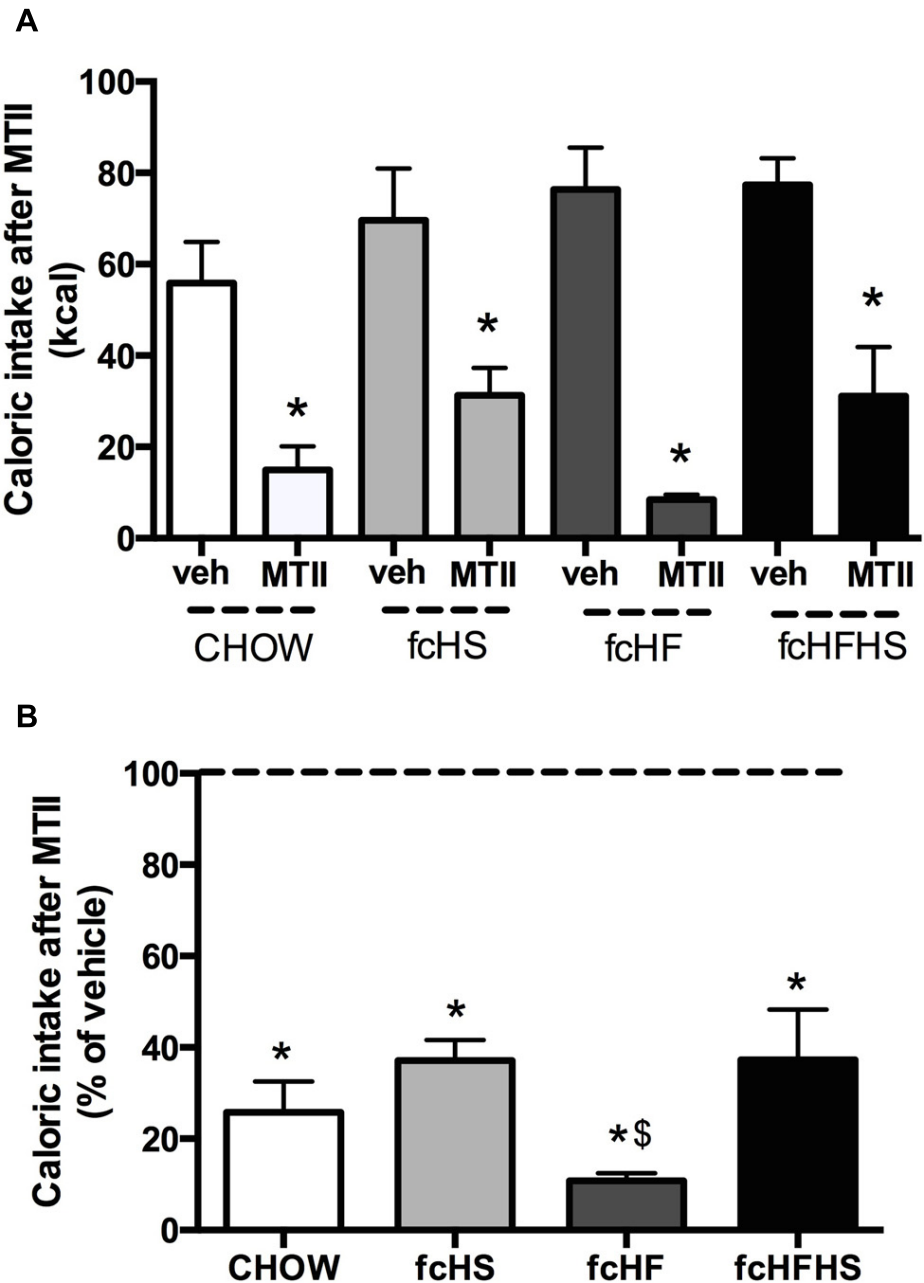
**Melanotan II (MTII) reduces caloric intake to a larger extent in rats on the fcHF diet.** Feeding response to MTII in rats on the CHOW, fcHS, fcHF, or fcHFHS diet. Absolute caloric intake **(A)** and as percentage of vehicle **(B)** 15 h after MTII ICV (0.3 nmol) upon 4 weeks diet exposure. Food intake was significantly inhibited in all diet groups. The extent of inhibition was significantly greater in fcHF rats. ∗Significantly different from vehicle injection in the same diet (*P* < 0.05). ^$^Significantly different from other diet groups (*P* < 0.05).

After 4 weeks diet exposure, caloric intake was determined 15 h after administration of MTII in the lateral ventricle. MTII decreased caloric intake in all diet groups (**Figure 1A**), but the inhibitory effect was significantly different between groups (**Figure 1B**). Two-way ANOVA on the raw data showed an effect of *Diet* [*F*(3,58) = 15.22; *P* < 0.01], *MTII* [*F*(1,58) = 11.88; *P* = 0.001], and *Interaction* [*F*(3,58) = 3.37; *P* = 0.025]. The percentage change from baseline vehicle was calculated and MTII was shown to significantly decrease food intake in all diet groups (**Figure 1B**). Additionally, significant differences in feeding responses to MTII between the groups were observed [*F*(3,30) = 3.1; *P* = 0.041], with food intake in rats on the fcHF diet being significantly lower than in the other diet groups (**Figure 1B**).

**FIGURE 2:**
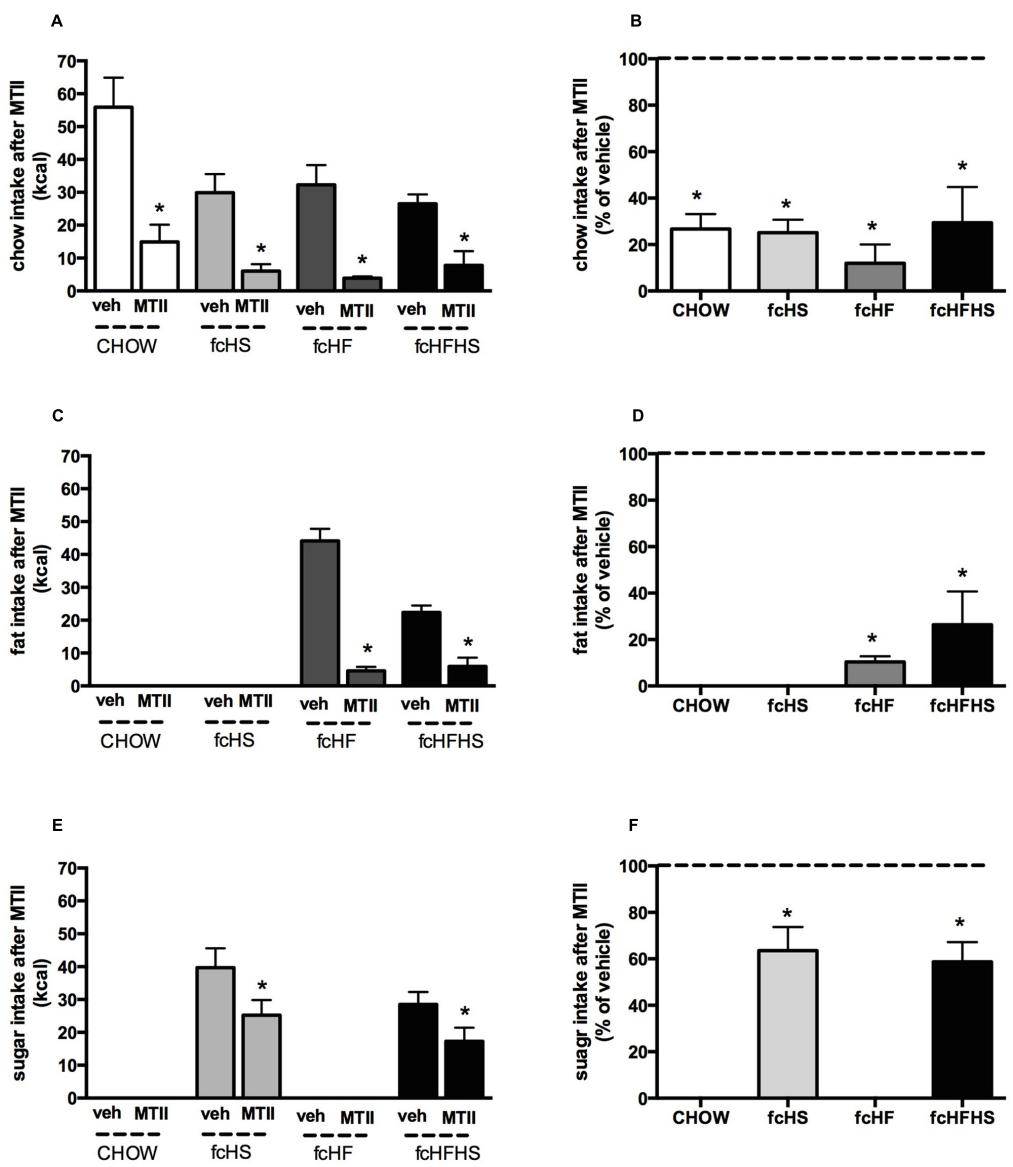
**Melanotan II (MTII) strongly reduces chow and fat content.** Effect of MTII on diet components chow **(A,B)**, fat **(C,D)**, and sugar **(E,F)** 15 h after MTII ICV (0.3 nmol) upon 4 weeks diet exposure depicted as absolute intake (kcal) **(A,C,E)** and as % of vehicle intake **(B,D,F)**. ∗Significantly different from vehicle injection in the same diet (*P* < 0.05).

MTII significantly decreased intake of the chow component in all diet groups (**Figure 2A**). The intake of chow after MTII (as percentage of vehicle) was 26 ± 6% (*P* < 0.01) in CHOW, 25 ± 6% (*P* < 0.01) in fcHS, 18 ± 8% (*P* < 0.05) in fcHF and 27 ± 15% (*P* < 0.01) in fcHFHS diet (**Figure 2B**) and was not significantly different between diet groups [*F*(3,20) = 0.22; *P* = 0.88]. MTII strongly inhibited fat intake in rats on the fcHF and fcHFHS diet (**Figure 2C**). The intake of fat after MTII (as percentage of vehicle) was 10 ± 2% (*P* < 0.001) in fcHF and 27 ± 14% (*P* < 0.001) in fcHFHS diet (**Figure 2D**) and was not significantly different between diet groups (*P* = 0.26). MTII also inhibited sugar intake in rats on the fcHFHS and fcHS diet (**Figure 2E**). The intake of sugar after MTII (as percentage of vehicle) was 65 ± 10% (*P* < 0.05) in fcHS and 59 ± 9% (*P* < 0.01) in fcHFHS diet (**Figure 2F**) and was not significantly different between diet groups (*P* = 0.68). One day after MTII administration, all groups significantly lost body weight. The body weight loss was not significantly different between diet groups (data not shown).

## Discussion

We here show that MTII inhibited caloric intake to a larger extent in rats on the fcHF diet compared to rats on the CHOW, fcHFHS, or fcHS diet. As rats on the fcHF diet lacked the sugar component and consumed more calories from fat compared to rats on the fcHFHS diet, and fat intake was the most inhibited after MTII administration, these data point toward an important role for diet composition and particularly the fat content in the diet in sensitivity to melanocortins. fcHF-fed rats had similar caloric intake, body weight, adiposity, and plasma leptin concentrations as rats on the fcHS diet (la Fleur et al., 2010; van den Heuvel et al., 2014b), indicating that the response to MTII is not explained by obesity. Moreover, rats on the fcHFHS diet showed increased obesity, fat mass, and leptin levels (la Fleur et al., 2010; van den Heuvel et al., 2014b), but did not show altered response to MTII compared to rats on the CHOW diet. Additionally, fcHS-fed rats did not show a reduced response to MTII, further indicating that the increased fat content is important for the enhanced response of MTII in rats on the fcHF diet. Collectively, these data show that the response of the melanocortin system is not explained by obesity but rather by diet composition and particularly by the fat content of the diet.

MTII inhibited all dietary components in rats on CHOW, fcHS, fcHF, and fcHFHS diet, although sugar intake was less inhibited compared to chow and saturated fat. This difference in inhibition by MTII of the three different components could also underlie the finding that total caloric intake was most reduced by MTII in rats on the fcHF diet, as they did not consume sugar. As the melanocortin system has been linked to preference for fat intake (Hagan et al., 2001; Samama et al., 2003), the enhanced total feeding inhibition to MTII in fcHF rats may be due to the increased fat content of the fcHF diet compared to the fcHFHS diet. Both the average percentual (49.6% fcHF vs. 26.7% fcHFHS) and the absolute (49.1 kcal/day fcHF vs. 29.7 kcal/day fcHFHS) basal intake of fat in rats on the fcHF diet is twice as high as the average in rats on fcHFHS diet, which supports the idea that the feeding response of the melanocortin system depends on diet composition and particularly on the basal fat intake (Kinzig et al., 2005; Morens et al., 2005).

The enhanced response in rats on the fcHF diet might also have been due to alterations in endogenous melanocortin signaling as a result of diet exposure (Harrold et al., 1999, 2000). Although in the current study we found that hypothalamic POMC and AgRP mRNA levels were not different after 4 weeks diet, we previously showed higher melanocortin receptor binding levels in fcHF compared to fcHFHS rats after 1 week diet (van den Heuvel et al., 2011). Therefore, changes in melanocortin receptor density may underlie the differential response to MTII between rats on the fcHF and on the fcHFHS diet, but we cannot link these to changes in production of their ligands. On the other hand, the differential feeding inhibition by MTII may be due to alterations in neuropeptides downstream of the receptor as for example, both galanin (Akabayashi et al., 1994; Odorizzi et al., 1999) and opioids (Hagan et al., 2001) have been proposed to be involved in the melanocortin-induced changes in fat intake. We here administered MTII in the lateral ventricle, which would reach all areas of the brain including those involved in reward (Samama et al., 2003). The specific effects of melanocortins on fat intake have also been observed after third ventricle as well as after local administration in several brain regions, including PVN and amygdala (Boghossian et al., 2010). Future research should investigate which brain areas and neuropeptides are involved in the enhanced response to MTII in fcHF-fed rats.

The fcHFHS diet represents several features of the human obesity situation including increased fat mass, insulin resistance and peripheral leptin resistance compared to rats on the CHOW, fcHF, and fcHF diet (la Fleur et al., 2011; van den Heuvel et al., 2014b). Moreover, as opposed to other animal models, rats on the fcHFHS diet also show specific human like characteristics such as persistent hyperphagia, snacking behavior (i.e., increased meal frequency without reducing meal size) and increased motivation behavior (even in sated animals; la Fleur et al., 2007, 2014; la Fleur and Serlie, 2014). Interestingly, sensitivity to MTII is not reduced in rats on the fcHFHS diet, as the feeding response to MTII was similar to CHOW, suggesting that the downstream targets are still active. This is in correspondence with other DIO models (Pierroz et al., 2002) and models of leptin resistance such as aging related and leptin-induced leptin resistance that also show normal functioning of the melanocortin pathway upon MTII treatment, despite the existence of leptin resistance (Scarpace et al., 2003; Zhang et al., 2004).

## Conclusion

Rather than the level of obesity, is the composition of the diet, in particular the fat content, important in the feeding responses of the melanocortin system.

## Conflict of Interest Statement

The authors declare that the research was conducted in the absence of any commercial or financial relationships that could be construed as a potential conflict of interest.
